# Disruption of a *Plasmodium falciparum* cyclic nucleotide phosphodiesterase gene causes aberrant gametogenesis

**DOI:** 10.1111/j.1365-2958.2008.06267.x

**Published:** 2008-05-13

**Authors:** Cathy J Taylor, Louisa McRobert, David A Baker

**Affiliations:** Department of Infectious and Tropical Diseases, London School of Hygiene & Tropical MedicineKeppel Street, London WC1E 7HT, UK

## Abstract

Phosphodiesterase (PDE) and guanylyl cyclase (GC) enzymes are key components of the cGMP signalling pathway and are encoded in the genome of *Plasmodium falciparum*. Here we investigate the role of specific GC and PDE isoforms in gamete formation – a process that is essential for malaria transmission and occurs in the *Anopheles* mosquito midgut following feeding on an infected individual. Details of the intracellular signalling events controlling development of the male and female gametes from their precursors (gametocytes) remain sparse in *P. falciparum*. Previous work involving the addition of pharmacological agents to gametocytes implicated cGMP in exflagellation – the emergence of highly motile, flagellated male gametes from the host red blood cell. In this study we show that decreased GC activity in parasites having undergone disruption of the *PfGCβ* gene had no significant effect on gametogenesis. By contrast, decreased cGMP-PDE activity during gametocyte development owing to disruption of the *PfPDEδ* gene, led to a severely reduced ability to undergo gametogenesis. This suggests that the concentration of cGMP must be maintained below a threshold in the developing gametocyte to allow subsequent differentiation to proceed normally. The data indicate that PfPDEδ plays a crucial role in regulating cGMP levels during sexual development.

## Introduction

Over 1 million deaths per year are caused by infection with the malaria parasite *Plasmodium falciparum*. Pathology is caused by asexually replicating forms that invade and develop inside red blood cells (RBC), whereas transmission of malaria requires a sexual phase of the life cycle involving formation of distinct male and female parasites (gametocytes) that also develop within host RBC. These gamete precursors are ingested by a female *Anopheles* mosquito during feeding on an infected individual. As a prerequisite to fertilization, the gametocytes must escape from their encapsulating RBC upon entering the mosquito midgut. *In vitro*, the first visible sign of differentiation is a transformation in gametocyte morphology from crescent-shaped to spherical (rounding up). Apart from emergence from the host RBC and parasitophorous vacuolar membrane, the female apparently undergoes few obvious morphological changes at this stage and is ready for fertilization. The male, by contrast, undergoes a series of spectacular metabolic and morphological changes. Three rounds of DNA replication and endomitotic division are accompanied by the assembly of eight axonemes and flagella in less than 10 min (Sinden *et al*., 1996; Alano and Billker, 2005), resulting in the release of eight highly motile male gametes that are visible under the light microscope. Though this process was first observed over a hundred years ago, significant advances in our understanding of these events at the molecular level in the ensuing years have been surprisingly rare. *In vitro*, exflagellation can be stimulated by a temperature decrease coupled with a bicarbonate ion-dependent rise in pH (Nijhout and Carter, 1978; Sinden, 1983). The pH change can be replaced by a mosquito derived factor (Nijhout, 1979) which was later discovered to be xanthurenic acid (XA) (Billker *et al*., 1998; Garcia *et al*., 1998). In *P. berghei*, XA triggers a rapid increase in intracellular Ca^2+^ which is thought to activate a Ca^2+^-dependent protein kinase (CDPK4) implicated in regulation of cell cycle progression in male gametocytes (Billker *et al*., 2004). Other second messenger systems have also been implicated in the process. The phospholipase C (PLC) inhibitor, neomycin, was shown to abolish exflagellation (Ogwan'g *et al*., 1993) and the formation of inositol (1,4,5) triphosphate and diacylglycerol was found to correlate with the initial events of flagellar development (Martin *et al*., 1994). Evidence of a role for the cGMP signalling pathway in exflagellation has also been presented (Kawamoto *et al*., 1990). It was reported that addition of cAMP had no effect on *P. berghei* or *P. falciparum* exflagellation and 1–10 mM caffeine (a cAMP-PDE inhibitor) inhibited exflagellation. However, at the non-permissive pH of 7.3, 10 mM IBMX (a cAMP/cGMP-PDE inhibitor) stimulated exflagellation (to 50% of the positive control stimulated at pH 7.8), suggesting that cGMP, but not cAMP, may be involved in the induction of exflagellation. Furthermore, addition of cGMP or nitroprusside (an activator of mammalian soluble GC) enhanced exflagellation levels in both species. Consistent with these results, addition of *N*-methyl-hydroxylamine (an inhibitor of soluble GC activation) inhibited exflagellation (Kawamoto *et al*., 1990). It has also been observed that addition of XA to gametocyte particulate fractions elevated GC activity (Muhia *et al*., 2001).

Genes likely to be involved in cyclic nucleotide signalling have recently been identified in *P. falciparum* (Carucci *et al*., 2000; Deng and Baker, 2002; Yuasa *et al*., 2005). Two unusual membrane-associated GCs (PfGCα and PfGCβ) are encoded in the *Plasmodium* genome and are expressed in *P. falciparum* gametocytes. They have an unusual, potentially bifunctional, structure with a C-terminal, paired cyclase catalytic domain and an N-terminal P-type ATPase-like domain. Although the predicted toplogy of the cyclase domain resembles eukaryotic G protein-dependent adenylyl cyclases, heterologous expression has confirmed the catalytic specificity of these molecules (Carucci *et al*., 2000). The *P. falciparum* genome encodes four putative cyclic nucleotide phosphodiesterases (PfPDEα-δ) and microarray analysis has shown their expression at the mRNA level is developmentally regulated (Young *et al*., 2005). The likely downstream effector of cGMP in *P. falciparum* is a single cGMP-dependent protein kinase (PfPKG) which has some structural and biochemical properties unique to apicomplexan parasites. In *P. falciparum*, PfPKG expression occurs in both the asexual and sexual blood stages of the life cycle (Deng and Baker, 2002; Diaz *et al*., 2006). Using an allelic replacement strategy, we have demonstrated an essential role for PKG in gametogenesis (McRobert *et al.*, in press).

Cyclic GMP mediates diverse functions across eukaryotic phyla ranging from motility in the ciliate protozoan *Paramecium* to colour vision in mammals. In the present study we aimed to investigate the role of cGMP in *P. falciparum* sexual development. We have used a genetic approach to examine the effects of interfering with cGMP synthesis or hydrolysis. A decrease in cGMP levels in gametocytes had no effect on subsequent development, but a premature increase in concentration owing to disruption of a cGMP-PDE gene severely reduced the ability of the parasite to differentiate.

## Results

### Disruption of a guanylyl cyclase gene (PfGCβ) leads to significantly reduced enzyme activity in gametocytes, but normal levels of gametogenesis are retained

**Fig. 1:**
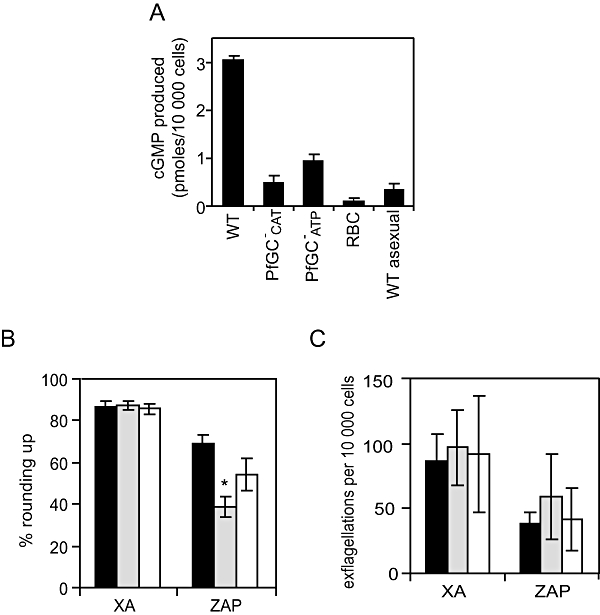
Phenotypic analysis of the PfGCβ^−^ parasites. A. Stage V gametocyte particulate fractions from WT and the two PfGCβ^−^ mutant clones (
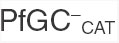
 and 
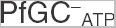
) were assayed for GC activity. Uninfected RBC and WT asexual blood stage particulate fractions were included as controls. The amount of cGMP produced by 10^4^ cells was measured using a 96-well plate cGMP competitive enzyme immunoassay system. The data shown are the mean of five individual assays from multiple parasite cultures carried out in triplicate, with error bars showing the standard error of the mean. B. Rounding up of WT (black bars), 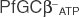 (grey bars) and 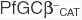 (white bars) gametocytes was measured after addition of either XA (100 μM) or zaprinast (400 μM). Ten minutes post stimulation, a minimum of 200 live cells were counted by light microscopy and scored as either round or gametocyte-shaped. Results show mean counts for a minimum of four experiments counted in duplicate, with error bars showing the standard error of the mean. The asterisk represents a statistically significant result. C. Exflagellation of WT (black bars), 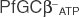 (grey bars) and 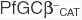 (white bars) male gametocytes was measured after addition of either XA (100 μM) or zaprinast (400 μM). Ten minutes post stimulation, cells were observed with a light microscope for 10 min and the number of centres of exflagellation scored per 10 000 cells. Results show mean counts for a minimum of three experiments counted in duplicate, with error bars showing the standard error of the mean.

There are two closely related GCs in *P. falciparum* (Carucci *et al*., 2000). The *PfGCα* gene is transcribed at low levels in both sexual and asexual blood stage parasites (Young *et al*., 2005) and it is refractory to deletion (as is its orthologue in *P. berghei*, D.A. Baker, A.F. Cowman and A.P. Waters, unpubl. data), suggesting that it is essential in the asexual blood stages. In contrast, *PfGCβ* is transcribed at relatively high levels in mature gametocytes but not in asexual blood stages, and was successfully disrupted in this study. Two independent cloned lines were generated using a pHTK-based transfection method, in which one of two essential functional domains of PfGCβ was replaced with the hDHFR sequence. 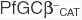 contains a deletion in the cyclase catalytic domain and 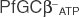 contains a deletion in the P-type ATPase-like domain (Fig. S1). The position of integration in each of the clones was verified by PCR, Southern blotting and pulsed-field gel electrophoresis (PFGE). In all clones tested, both asexual replication and the rate of conversion to gametocytes were equivalent to levels observed in control cell lines (data not shown). Mature stage V gametocyte particulate fractions extracted from the mutant and control cell lines were assayed for GC activity at 30°C and the resulting cGMP levels were measured. In both of the mutant lines, levels of GC activity were significantly lower than in control wild-type (WT) gametocytes (approximately three to sixfold less cGMP synthesis measured per 10 000 cells) (Fig. 1A), indicating that disruption of either of the functional domains of PfGCβ can ablate its GC catalytic activity. It is assumed that residual activity is derived from PfGCα which is also expressed in gametocytes.

Next, the mutants were assayed for their ability to round up and exflagellate. While both of the PfGCβ^−^ mutant cell lines retained the ability to round up and exflagellate at normal levels upon addition of 20 μM XA (Fig. 1B and C), they consistently showed a reduced responsiveness to zaprinast in terms of rounding up (Fig. 1B). We have shown previously that zaprinast (a cGMP-PDE) can stimulate rounding up and gametogenesis in the absence of XA or a pH increase (McRobert *et al.*, in press). This result suggests that the reduced cGMP synthesis accompanying disruption of PfGCβ cannot be fully compensated by inhibition of cGMP hydrolysis by zaprinast. However, this trend was significant for only one of the clones (*P* = 0.0012) over the course of nine replicate experiments. Levels of stimulation of exflagellation by zaprinast in mutants were not significantly different (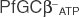 *P* = 0.603; 
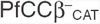
 *P* = 0.332) to those in controls (Fig. 1C). Together the data show that gametogenesis can proceed in the absence of PfGCβ activity.

### PfPDEδ^−^ mutants have significantly reduced cGMP-PDE activity and a greatly reduced ability to undergo gametogenesis

**Fig. 2:**
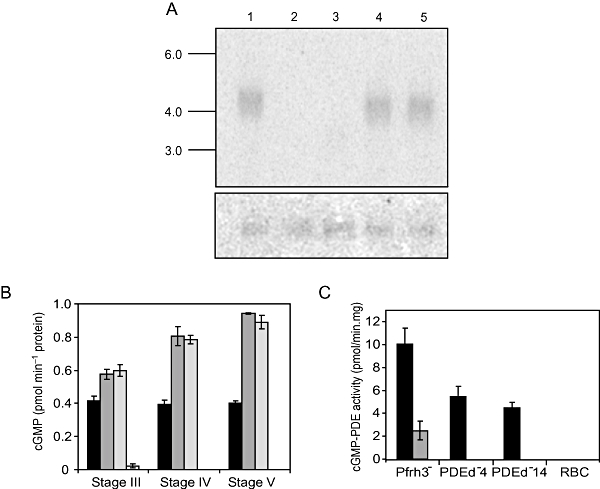
cGMP-PDE activity in PfPDEδ^−^ parasites. A. A northern blot containing total RNA (4 μg in each lane) from PfPDEδ^−^, PfPDEγ^−^ clones and control Pfrh3^−^ parasites. The blot was probed with the catalytic domain of PfPDEδ (upper panel) and the membrane was stripped and reprobed with a region of the *P. falciparum* glycerol kinase gene (PlasmoDB identifier PF13_0269) (lower panel) to demonstrate equal loading. Lane 1, Pfrh3^−^ RNA; lanes 2 and 3, PfPDEδ^−^ clones 4 and 14 respectively; lanes 4 and 5, PfPDEγ^−^ clones A3 and A4 respectively. Sizes in kilobases are indicated to the left. B. The total intracellular cGMP concentration was measured in stages III to V gametocytes in PfPDEδ^−^ clones and controls using an enzyme immunoassay based on a competition assay. Black bars denote control (Pfrh3^−^) cells, PfPDEδ^−^clones are represented by dark grey (clone 4) and light grey (clone 14) bars. The white bar represents the cGMP content of uninfected erythrocytes. Gametocytes were harvested using Nycodenz and aliquots of 10^7^ cells were assayed in triplicate wells. The assay was carried out three times and a representative result is shown. Error bars denote the standard error of the mean. C. cGMP-PDE activity was measured in particulate fractions of stage V gametocytes from PfPDEδ^−^ clones (PDEd^−^4 and PDEd^−^14) and Pfrh3^−^ controls. Fractions were assayed in the absence (black bars) and presence (grey bars) of 400 μM zaprinast. Gametocytes were harvested using Nycodenz and aliquots of 10^7^ cells were assayed in triplicate wells. The results are the mean of three independent experiments. RBC, uninfected red blood cells. Error bars represent the standard error of the mean.

There are four putative PDE genes in the *P. falciparum* genome which we have designated *PfPDEα-δ* (PlasmoDB identifiers: PFL0475w, MAL13P1.118, MAL13P1.119 and PF14_0672 respectively; http://plasmodb.org/plasmo/). The cyclic nucleotide specificity of the four encoded enzymes cannot be predicted on the basis of primary amino acid sequence. The relatively high level of *PfPDEδ* mRNA expression (compared with the other three PfPDEs) in developing and mature gametocytes corresponds with significant levels of cGMP-PDE activity and barely detectable cAMP-PDE activity in the same life cycle stage (R.M. Cummings, C.J. Taylor and D.A. Baker, unpubl. data). We therefore reasoned that PfPDEδ is capable of cGMP hydrolysis. To investigate the role of PfPDEδ in sexual development, and the effect of interfering with cyclic nucleotide hydrolysis in the parasite, we disrupted *PfPDEδ*. Following transfection and drug selection, several cloned lines were generated and two of these were selected for detailed analysis. *PfPDEδ* was disrupted by a complex double cross-over event just upstream of the translational start codon in one of the lines (clone 4) and by a 3′ single cross-over event in the second (clone 14; Fig. S2). Northern blotting confirmed the absence of *PfPDEδ* mRNA expression in both of the cloned lines (Fig. 2A). In these clones both asexual replication and the rate of conversion to gametocytes were equivalent to levels observed in control cell lines (data not shown). The control was a transfected 3D7 line, generated in parallel, containing a gene disruption in a locus (*Pfrh3*) previously reported to be a pseudogene (Duraisingh *et al*., 2002). Gametocytes from PfPDEδ^−^ mutants and controls were harvested and used to measure differences in total cellular cGMP levels and native PDE activity. Intracellular levels of cGMP were significantly higher in independent PfPDEδ^−^ mutants (stage III-V) compared with control cell lines (Fig. 2B) and increased to approximately twofold higher in stage V gametocytes. Consistent with these measurements, gametocyte (stage V) particulate fractions from PfPDEδ^−^ clones demonstrated decreased levels (approximately 50%) of *in vitro* cGMP-PDE activity compared with control cell lines (Fig. 2C, black bars). Measurements of cAMP-PDE activity were also carried out and consistently low levels were obtained that were indistinguishable from levels measured in controls (approximately 1 pmol min^−1^ (mg protein)^−1^ in all cases, data not shown). Together, these data provide biochemical evidence that PfPDEδ has cGMP-PDE catalytic activity. Interestingly, the cGMP-PDE levels in the PfPDEδ^−^ mutant gametocyte particulate fractions were reduced rather than removed completely (Fig. 2C). This indicates that a second cGMP-PDE is expressed (or possibly upregulated as a result of the KO event) at this life cycle stage. To investigate the residual activity further, we tested the effect of zaprinast on the cGMP-PDE activity of PfPDEδ^−^ mutant and control cell lines. Addition of excess zaprinast to control particulate fractions reduced cGMP-PDE activity by approximately 75% whereas when added to mutant fractions it totally removed activity (Fig. 2C, grey bars). This strongly suggests that PfPDEδ is relatively insensitive to zaprinast whereas the residual activity is zaprinast-sensitive.

**Fig. 3:**
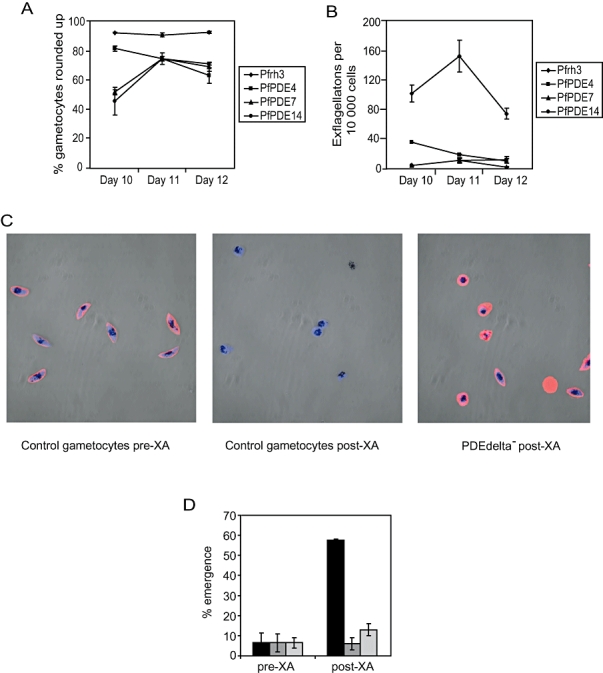
Rounding up and emergence levels in PfPDEδ^−^ parasites. A. The percentage of rounded up gametocytes in three independent PfPDEδ^−^ clones (PfPDE4, 7 and 14) and Pfrh3^−^ controls (Pfrh3) was evaluated 10 min after the addition of 20 μM XA by counting 200 live gametocytes and scoring them as round or gametocyte-shaped. Results are based on three independent counts of each clone on three consecutive days (days 10–12). Error bars represent the standard error of the mean. B. The number of centres of exflagellation in the three independent PfPDEδ^−^ clones and Pfrh3^−^ controls was evaluated under the same conditions. Results are based on three independent counts of each clone on three consecutive days. Error bars represent the standard error of the mean. C. Merged confocal immunofluorescence images of Pfrh3^−^ control stage V gametocytes before (pre-XA, left panel) and after (post-XA, centre panel) addition of 20 μM XA and PfPDEδ^−^ stage V gametocytes (PDEdelta^−^ post-XA) after (right panel) addition of 20 μM XA. Parasites were fixed with paraformaldehyde and stained with a human Band 3 antibody (red) to determine whether parasites had emerged from their host RBC. Blue shows DAPI stain. D. The percentage of emergence was evaluated by counting reactivity of cells with a Band 3 antibody in two independent PfPDEδ^−^ clones and Pfrh3^−^ controls before and after addition of 20 μM XA. Black bars denote control (Pfrh3^−^) cells, PfPDEδ^−^clones are represented by dark grey (clone 4) and light grey (clone 14) bars. Results are based on duplicate independent counts from the same flask of gametocytes on a single day. Error bars represent the standard error of the mean.

While PfPDEδ^−^ mutants produced morphologically normal gametocytes up to and including stage V of development, a pronounced difference was observed upon stimulation of gametogenesis. The ability of the PfPDEδ^−^ mutant cell lines to round up in the presence of XA was reduced significantly compared with controls (Fig. 3A) and levels of exflagellation were reduced drastically (Fig. 3B). The ability of mutants and controls to round up and exflagellate was measured on three consecutive days (in three independent clones) to confirm the dramatic decrease in the ability of the mutants to undergo gametogenesis (Fig. 3A and B). This effect was reproduced when zaprinast was used in place of XA, confirming that the normal signalling pathways triggering exflagellation were not fully functional in these clones.

An antibody to the abundant RBC anion exchanger Band 3 was used in IFA (as a marker for the presence of human RBC) to examine the ability of activated mutant gametocytes to emerge from RBC. Prior to activation, Pfrh3^−^ control gametocytes react strongly with the antibody (Fig. 3C, left panel), indicating that parasites are fully enclosed within the RBC. Upon emergence of control gametocytes after stimulation with XA, no reaction with the antibody is observed (Fig. 3C, centre panel), indicating successful escape from the host red cell. Figure 3C (right panel) shows a representative field containing PfPDEδ^−^ gametocytes after stimulation with XA; the host red cell membrane is still in tact despite activation of gametogenesis. The proportion of emerged parasites assessed by reaction with the Band 3 antibody in both control and mutant lines is shown in Fig. 3D. The emergence of PfPDEδ^−^ mutants was almost unchanged after stimulation (Fig. 3C, right panel and Fig. 3D). This indicates that the majority of PfPDEδ^−^ gametocytes fail to emerge from the host red cell upon addition of XA. Taken together, these data suggest that PfPDEδ^−^ cells are exposed to high levels of cGMP during gametocyte development and that this is extremely deleterious to the ability of parasites to undergo gametogenesis.

## Discussion

It is known that a decrease in temperature combined with either a mosquito-derived factor (XA) or a rise in pH are required for *in vitro* stimulation of gametogenesis in *Plasmodium*. Independent lines of evidence have suggested a role for the cGMP signalling pathway in regulating this process (McRobert *et al.*, in press); (Kawamoto *et al*., 1990; Kawamoto *et al*., 1993; Muhia *et al*., 2001). This led us to hypothesize that elevated levels of endogenous cGMP might trigger these events. Here we have examined this question using a direct genetic approach deleting genes involved in the synthesis and hydrolysis of cGMP. Our results suggest that a reduced level of cGMP in gametocytes (owing to disruption of a GC gene) has little observable effect on gametocyte maturation or gametogenesis, but that a premature increase in cGMP levels (owing to disruption of a cGMP-PDE gene) is highly deleterious.

*A priori*, disruption of genes encoding a cGMP-PDE or a GC provides a means of testing the effects of increasing or decreasing cellular levels of cGMP respectively. In this study we found that asexual replication and gametocyte development are apparently normal in clones where *PfPDEδ* has been disrupted. However, mature gametocytes derived from these clones had significantly reduced cGMP-PDE activity and corresponding elevated cGMP levels. This correlated with a greatly decreased ability to undergo rounding up and exflagellation. The phenotype was displayed in independent clones in which the gene had been disrupted by entirely different integration events. A control line in which a pseudogene *Pfrh3* was deleted in a parallel transfection and drug selection experiment using the same parental stock of clone 3D7 retained normal levels of PDE activity and gametogenesis. Importantly, parasites generated in parallel using the same strategy but with a deletion of a second PDE gene [*PfPDEγ*, which is transcribed at low levels in gametocytes (Young *et al*., 2005)] also showed normal levels of rounding up and exflagellation. The *PfPDEγ*^−^ mutants showed no change in their ability to hydrolyse cAMP or cGMP in gametocytes. This further supports the specificity of the PfPDEδ^−^ phenotype; indicating that abnormally high levels of cGMP in maturing gametocytes interfere significantly with their ability to undergo gametogenesis. Attempts at complementation of PfPDEδ^−^ have so far not led to selection of viable parasites – a finding which may reflect the limitation of our knowledge of promoter sequences in malaria parasites.

To further define the point at which gametogenesis is blocked in the PfPDEδ^−^ clones, we used IFA with a RBC Band 3 antibody to investigate their ability to emerge from the RBC upon activation. The low proportion of emergence suggested strongly that both male and female gametocytes were affected. The effect of this observation in combination with the reduced level of exflagellation on transmission to the mosquito has yet to be determined, but it is likely that oocysts would either be very low in number or absent. It appears that a premature increase in cGMP levels has adverse consequences and that regulation of cGMP in gametocytes, at the level of its breakdown, is of critical importance. It has been reported that PfPKG is expressed in gametocytes at both the mRNA and protein level (Deng and Baker, 2002; Diaz *et al*., 2006). It is therefore possible that abnormally elevated cGMP levels (owing to disruption of PfPDEδ) may result in premature activation of PKG, leading to severe effects at the onset of gametogenesis. However, a transient increase in cGMP levels in fully mature WT gametocytes (caused by addition of zaprinast) can stimulate gametogenesis, indicating that the timing and perhaps duration of an increase in cGMP levels is crucial. The absence of PfPDEδ expression in mutants is also likely to lead to prolonged high levels of cGMP (and PKG activation).

Disruption of PfPDEδ reduced, but did not remove, native cGMP-PDE activity in gametocytes. This suggests that at least one of the other three PfPDE genes is expressed in the PfPDEδ^−^ clones during gametocyte development. This residual cGMP-PDE activity could be distinguished from that of PfPDEδ on the basis of its sensitivity to zaprinast. It is interesting that a highly conserved glycine residue implicated in interaction with cGMP and which defines zaprinast sensitivity in bovine PDE5 (Turko *et al*., 1998; Turko *et al*., 1999) is conserved in the other three PfPDEs, but not in PfPDEδ (K_640_). This supports the assertion that PfPDEδ may be relatively insensitive to zaprinast. Based on expression of the catalytic domain in *Escherichia coli*, it has been reported that PfPDEα (referred to as PfPDE1 in Yuasa *et al*., 2005) is a cGMP-specific PDE whose activity is inhibited by zaprinast with an IC_50_ of 3.8 μM. Mixed asexual blood stage membrane fractions were reported to have cGMP-PDE activity that was inhibited by zaprinast (IC_50_ 4.1 μM) at levels consistent with our own measurements (McRobert *et al*., in press). Zaprinast was also reported to inhibit asexual blood stage growth with an ED_50_ of 35 μM. As PfPDEα (PfPDE1) is a cGMP-PDE and is also transcribed at detectable levels in gametocytes, it is therefore a leading candidate for the zaprinast-sensitive cGMP-PDE activity expressed in the PfPDEδ^−^ clones. However, expression of *PfPDEδ* mRNA (unlike that of the other three *PfPDE* genes) rises steeply during gametocyte development, and our results show that it is not possible for the residual cGMP-PDE activity to compensate for the absence of PfPDEδ and leads to abnormally high levels of cGMP. We hypothesize that in WT gametocytes XA (in combination with reduced temperature) stimulates GC activity directly or indirectly and that the resultant rise in cGMP levels above a threshold activates PfPKG which in turn triggers gametogenesis. It is possible that this increase in cGMP concentration (or PKG activity) stimulates PfPDEδ which returns cGMP levels to a subthreshold concentration. In the *PfPDEδ* knockout cells, this control process breaks down; cGMP levels remain high throughout gametocyte development and this interferes with the ability of the parasite to undergo gametogenesis. Attempts to examine sex-specific effects of gene deletion were unsuccessful owing to the lack of specificity of reported male- and female-specific antisera in our hands.

In contrast to the findings above, normal levels of both rounding up and exflagellation were observed on addition of XA to PfGCβ^−^ gametocytes, despite their decreased ability to synthesize cGMP. It is feasible that there is redundancy of function between the two *P. falciparum* GCs as they are closely related in terms of amino acid sequence identity and predicted topology. There is also evidence that both are expressed in gametocytes at the mRNA and protein levels (Carucci *et al*., 2000; http://plasmodb.org/plasmo/). *PfGCα* (and its *P. berghei* orthologue) is refractory to deletion, suggesting that the gene may be essential in asexual blood stage parasites where it is known to be expressed (http://plasmodb.org/plasmo/). The only alternative source of cGMP synthesis is the RBC, although the level of cGMP in uninfected RBC has been shown previously to be very low (0.02 ± 0.01 pmol/10^7^ cells; Ghigo *et al*., 1995), consistent with our own results. Deletion of *PfGCβ* does not have detectable adverse effects on *in vitro* asexual replication, gametocyte development or XA-stimulated gametogenesis. However, the apparent reduced responsiveness to zaprinast could reflect the decreased ability of these cell lines to synthesize cGMP, where the absence of PfGCβ may not be fully compensated by inhibition of cGMP hydrolysis by zaprinast.

The GCβ orthologue has recently been deleted in *P. berghei* (Hirai *et al*., 2006). Consistent with our results in *P. falciparum*, sexual blood stage development and exflagellation were able to proceed in the PbGCβ^−^ mutants upon XA stimulation. However, the *in vitro* motility of the ookinete (that is required for invasion of the mosquito midgut wall) was severely compromised in these lines, suggesting an essential role for cGMP in this process. Furthermore, this suggests that expression of *PbGCα* cannot compensate for deletion of *PbGCβ* in the ookinete stage of the life cycle. Ookinete cultivation in *P. falciparum* is not possible.

We have used a direct genetic approach to delete genes involved in the synthesis and hydrolysis of cGMP. We demonstrate that a reduced level of cGMP in gametocytes resulting from the deletion of *PfGCβ* has little or no observable effect on gametocyte maturation or gametogenesis, but that increased levels of cGMP owing to *PfPDEδ* deletion result in an aberrant phenotype. Future work will focus on the potential role of the other three PfPDEs in gametogenesis; it will be of interest to determine the responsiveness to zaprinast in mutants lacking one or more of these genes. Investigation of the potential regulatory role of the N-terminal domain of the PfPDEs predicted to contain multiple transmembrane helices will also be important in understanding how cyclic nucleotides interact with other signalling pathways to control differentiation. It will also be of interest to further explore the role of cGMP in the post-fertilization stages of *P. falciparum* development. This work has provided genetic evidence that cGMP plays an important role in sexual development of the malaria parasite and could lead to novel ways of interfering with its transmission from human to mosquito.

## Experimental procedures

### Cell culture and gametogenesis assays

*Plasmodium falciparum* gametocytes were produced from the 3D7 clone in human A+ erythrocytes with 10% human serum (National Blood Service, UK), and enriched using Nycodenz (Nycoprep 1.077, Axis-Shield) centrifugation as previously described (Fivelman *et al*., 2007). For gametogenesis, cells were re-suspended at 5 × 10^6^ parasites per ml and induced with either 20 μM XA or 25–400 μM zaprinast in complete medium, and a reduction (of > 5°C) in temperature. Ten minutes post induction, cells were mounted on a wet smear. Rounding up was observed for a total of 200 cells, and exflagellation for a total of 10 000 cells over 10 min. Zaprinast was re-suspended in DMSO as a stock solution and diluted in complete medium for gametogenesis assays, while the XA stock was re-suspended in RPMI1640. The maximum final DMSO concentration in the gametogenesis medium was 0.4% which was used as a negative control.

### Generation of transgenic parasites and genotype analysis

Genetic manipulation of 3D7 was achieved by transfection with pHTK-based plasmids (Duraisingh *et al*., 2002). Two 1 kb fragments of the target gene were inserted into the vector at either end of the *dhfr* cassette to facilitate gene replacement. Transfection of *P. falciparum* 3D7 parasites was carried out following a method adapted from that of Deitsch *et al*. (2001). After transfection, parasites were returned to culture at 5% haematocrit, and parasitaemia was determined after 24 h. Positive selection with 5 nM WR99210 was initiated after 48 h. Transformants were subjected to two cycles of on-off WR selection and finally ganciclovir was applied at a concentration of 20 μM. Parasite lines were cloned by limiting dilution. Genotyping of cell lines and clones was carried out by a combination of PCR using primer pairs diagnostic for integration and hybridization with both genomic Southern blots and PFGE blots. These methods and northern blotting were carried out according to standard procedures (Sambrook *et al*., 1989).

### PDE assays

Phosphodiesterase activity in native parasite fractions was measured using a modification of a previously published method (Kuwayama *et al*., 2001). Parasites were frozen in liquid nitrogen and stored at −80°C until use. Parasites were re-suspended in 500 μl lysis buffer (20 mM hepes and 250 mM sucrose, pH 7.0), subjected to five cycles of freeze-thaw in liquid nitrogen and pelleted at 100 000 *g* for 30 min. Particulate fractions were re-suspended in lysis buffer containing EDTA-free protease inhibitors (Roche). PDE assays were carried out in triplicate wells of a 96-well plate in the presence of [^3^H]-labelled cGMP (GE Healthcare) for 30 min at 37°C. Reactions were terminated by boiling the plate for 1 min, followed by a 3 min centrifugation at 900 *g*. 1 unit of alkaline phosphatase was added to each well and incubated for 30 min at 37°C. [^3^H]-labelled guanosine was separated from the radiolabelled cGMP substrate using ion exchange (Bio-Rad AG® 1 × 8 resin). Supernatants containing the [^3^H]-labelled guanosine product were added to scintillation fluid (Optiphase Supermix, Wallac). Scintillation was measured using a Wallac 1450 Microbeta Liquid Scintillation Counter (Perkin Elmer) and PDE activity was expressed in pmol cGMP min^−1^ (mg protein)^−1^. Inhibition assays were carried out in the presence of compounds dissolved in DMSO. PDE assays were carried out at a native lysate dilution that gave 30% cGMP/cAMP hydrolysis.

### GC assays

Guanylyl cyclase activity in native parasite membrane fractions was measured using a modification of a previously published method (Muhia *et al*., 2001). Parasites were frozen in liquid nitrogen and stored at −80°C until use. Parasites were re-suspended in 500 μl lysis buffer (20 mM hepes and 250 mM sucrose, pH 7.0), subjected to five cycles of freeze-thaw in liquid nitrogen, passed through gauge 21 needles and pelleted at 100 000 *g* for 30 min. Membrane fractions were re-suspended in lysis buffer containing EDTA-free protease inhibitors (Roche) and 20 μl aliquots were added to 30 μl of reaction buffer (30 mM Tris, pH 8.0, 3 mM GTP, 16 mM theophylline) in triplicate and assayed for GC activity at 30°C for 30 min. The reactions were stopped by adding 30 μl of 0.5 M EDTA followed by boiling for 2 min. Samples were stored at −20°C prior to cGMP assay. Levels of cGMP were measured using a Biotrak cGMP competitive enzyme immunoassay kit (GE Healthcare) according to the manufacturer's instructions using the acetylation protocol. This enzyme immunoassay was also used independently for determination of intracellular cGMP levels in PDEδ^−^ mutants, without the GC step described above.

### Preparation of cells for IFA

For IFA 3 × 10^6^ mature *P. falciparum* gametocytes were fixed in paraformaldehyde according to a method used for *P. berghei* (Billker *et al*., 2002). Cells were washed thoroughly and allowed to settle on wells coated with 0.01% poly-l-lysine solution. Primary antibodies, either mouse anti-α tubulin (Tat-1; a gift from Keith Gull, University of Oxford) or mouse monoclonal anti-Band 3 (Abcam) were used at a concentration of 1:50, followed by a 1:10 000 dilution of Alexa Fluor 594 goat anti-mouse IgG (Molecular Probes). Vectashield (Vector Laboratories) containing DAPI allowed the cells to be visualized using a Zeiss Axovert LSM 510 confocal microscope. Images were captured using LSM 510 software (Carl Zeiss MicroImaging).

## Supplementary material

This material is available as part of the online article from: http://www.blackwell-synergy.com/doi/abs/10.1111/

j.1365-2958.2008.06267.x

(This link will take you to the article abstract).
