# Supplementary material for: Disruption of a Plasmodium falciparum cyclic nucleotide phosphodiesterase gene causes aberrant gametogenesis
Source: Mol Microbiol. 2008 May 13;69(1):110–8. doi: 10.1111/j.1365-2958.2008.06267.x (PMC2615252; doi:10.1111/j.1365-2958.2008.06267.x)
Supplement: Supplementary file 1 [file mmi0069-0110-SD1.pdf]

Supplementary Figure 1A

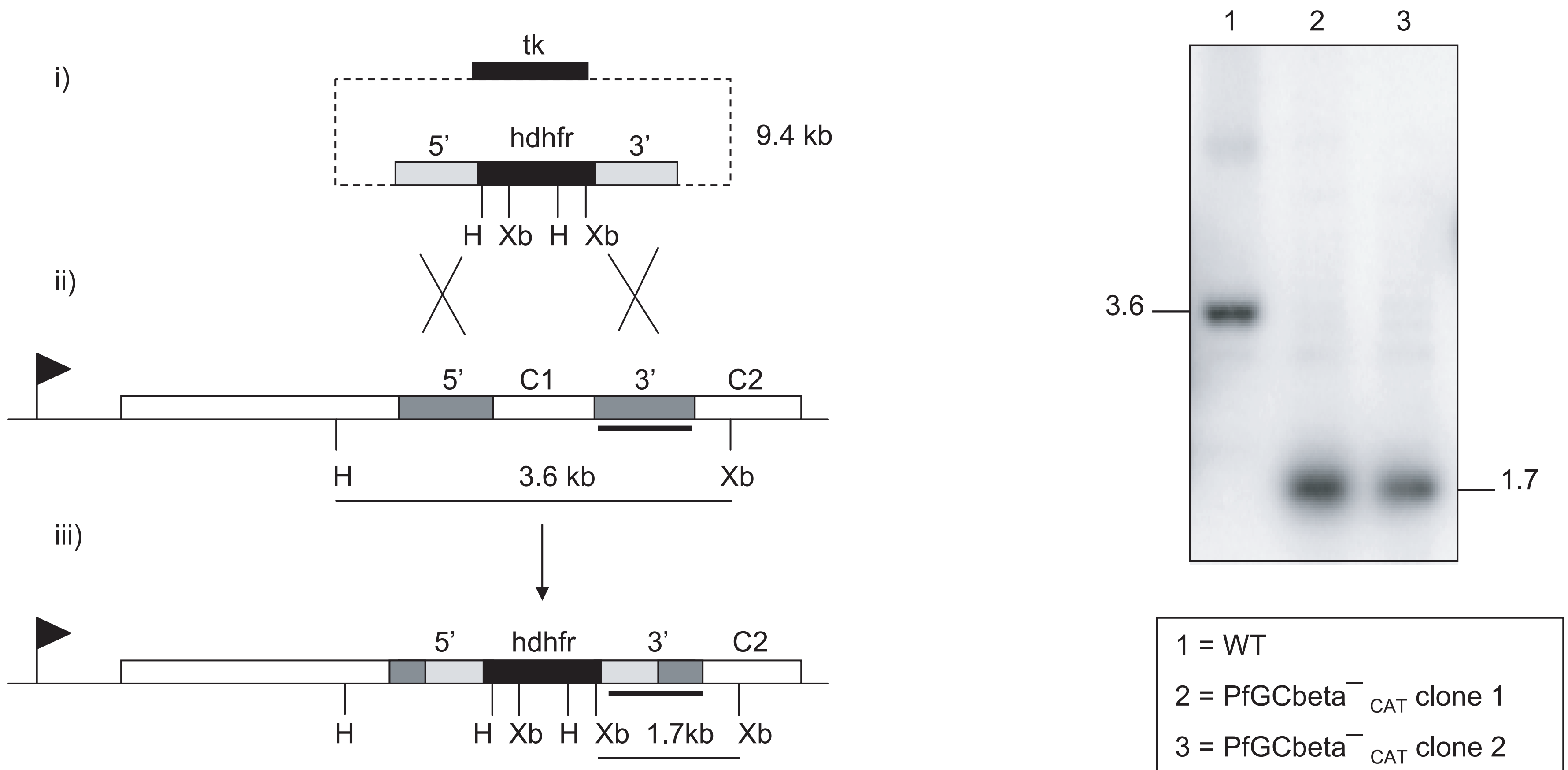

1 = WT

2 = PfGCbeta^{-} $_{\text{CAT}}$ clone 1

3 = PfGCbeta^{-} $_{\text{CAT}}$ clone 2

*Hin*dIII/*Xba* I digest

Probe = 3’ flank

i) Plasmid; ii) WT locus; iii) double crossover integration event found in clone 1 and clone 2

Introns not shown

Clone 1 used in all experiments

Supplementary Figure 1B

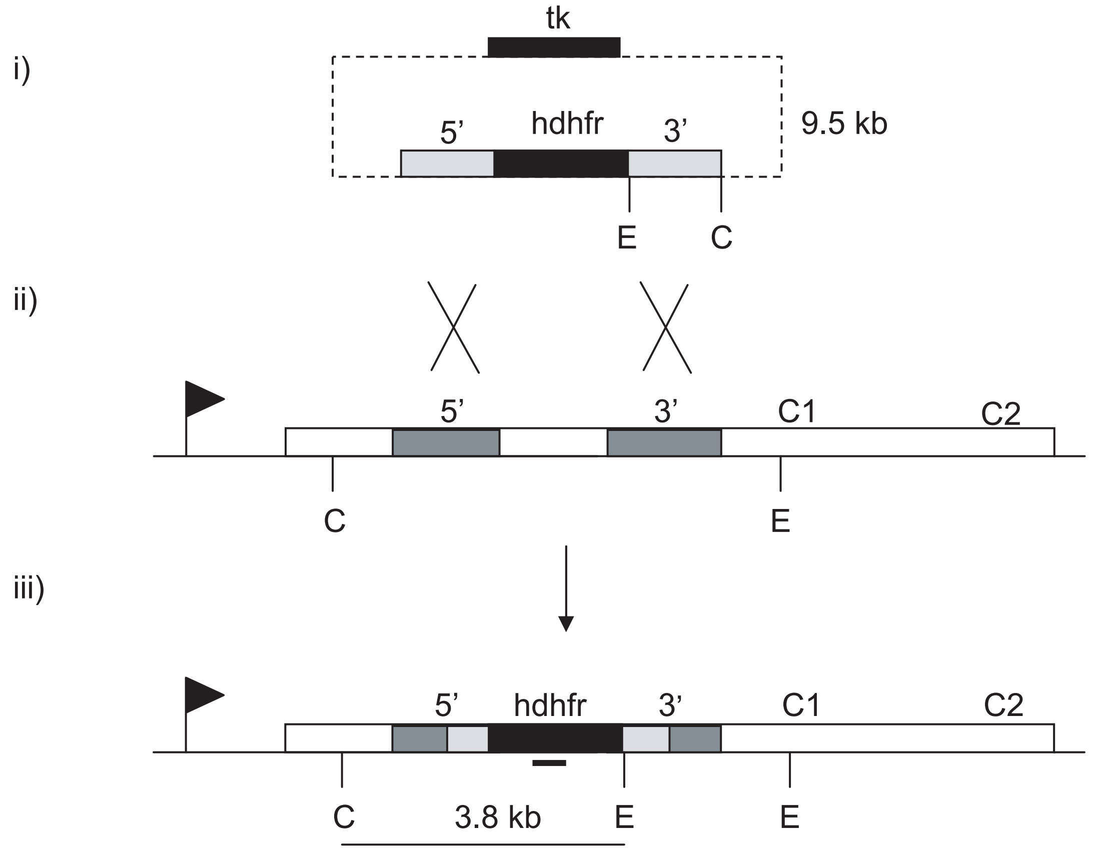

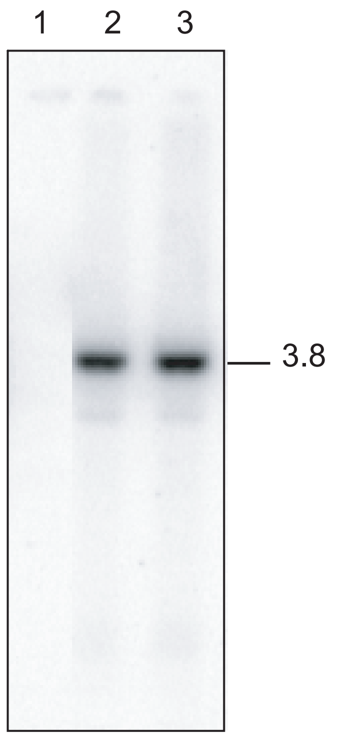

1 = WT

2 = PfGCbeta$^{-}_{ATP}$ clone 1

3 = PfGCbeta$^{-}_{ATP}$ clone 2

*Eco*RI/*Cla* I digest

Probe = hdhfr

i) Plasmid; ii) WT locus; iii) double crossover integration event found in clone 1 and clone 2

Introns not shown

Clone 1 used in all experiments

Supplementary Figure 2A

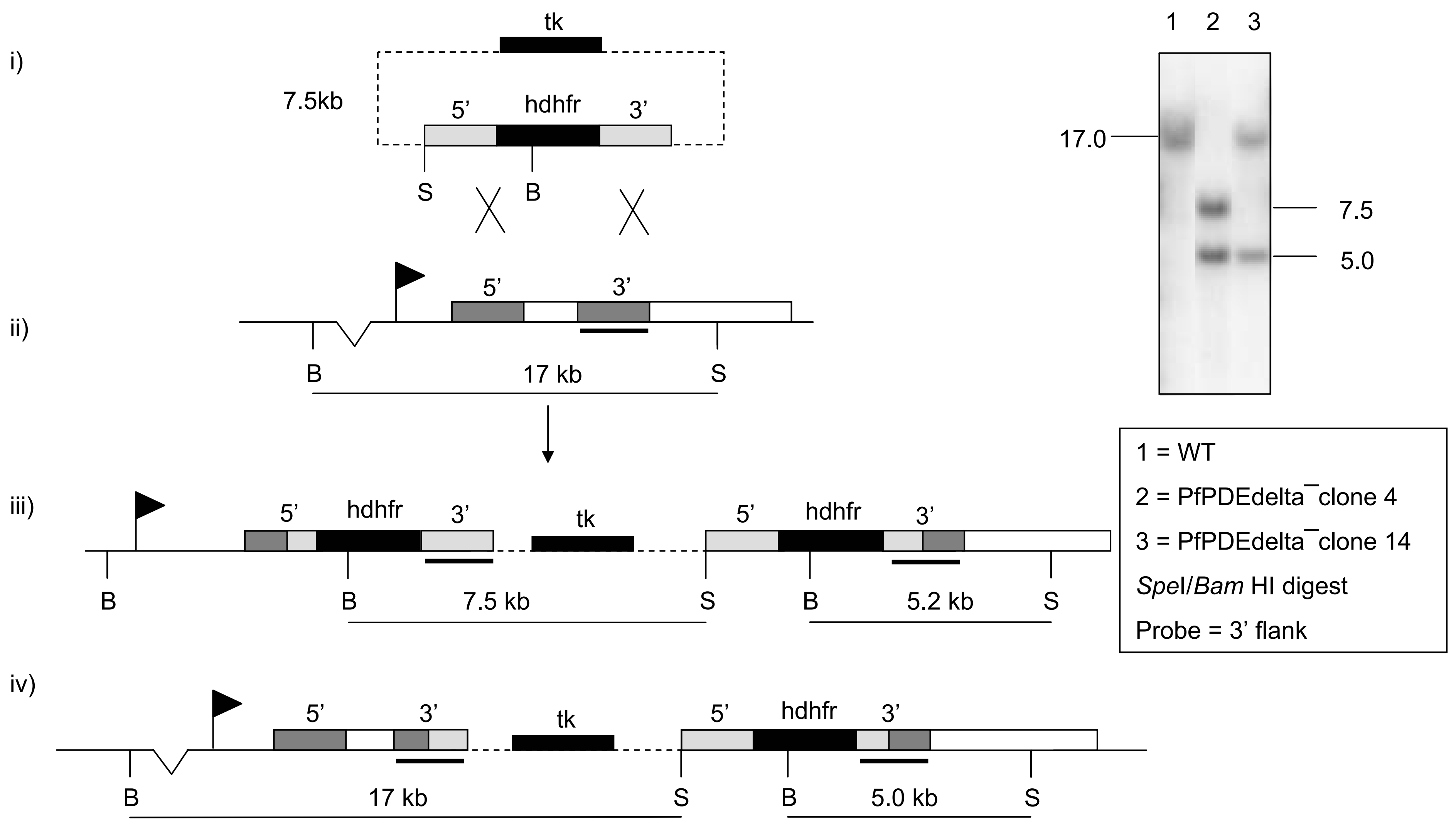

i) Plasmid, ii) wild type, iii) KO clone 4, iv) KO clone 14

Supplementary Figure 2B

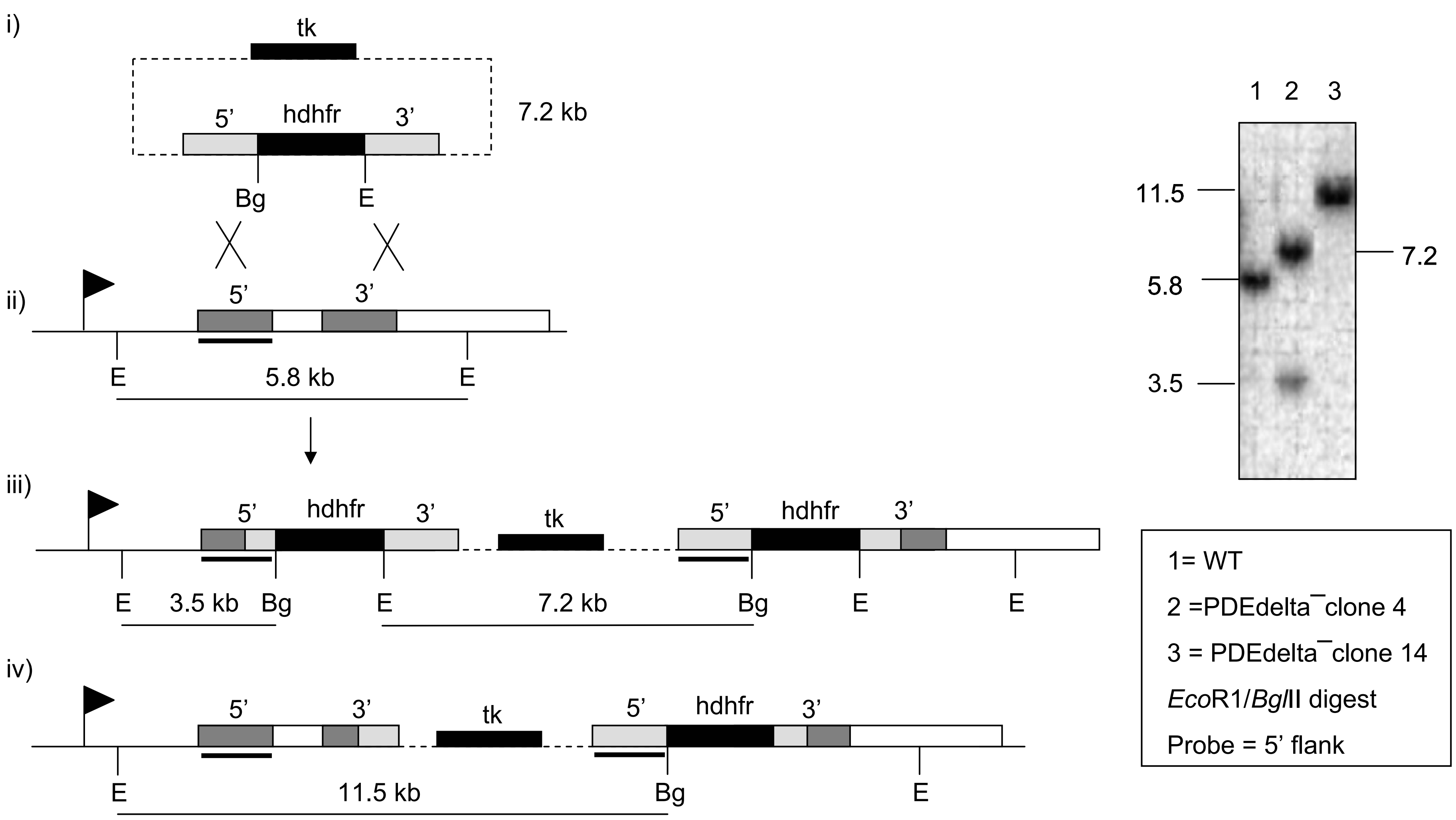

i) Plasmid, ii) wild type, iii) KO clone 4, iv) KO clone 14
